# Planarian shows decision-making behavior in response to multiple stimuli by integrative brain function

**DOI:** 10.1186/s40851-014-0010-z

**Published:** 2015-02-01

**Authors:** Takeshi Inoue, Hajime Hoshino, Taiga Yamashita, Seira Shimoyama, Kiyokazu Agata

**Affiliations:** Department of Biophysics, Graduate School of Science, Kyoto University, Kitashirakawa-Oiwake, Sakyo-ku, Kyoto 606-8502 Japan

**Keywords:** Planarian, Chemotaxis, Thigmotaxis, Thigmokinesis, Higher brain function, Decision-making, Integration, RNAi, Readyknock

## Abstract

**Introduction:**

Planarians belong to an evolutionarily early group of organisms that possess a central nervous system including a well-organized brain with a simple architecture but many types of neurons. Planarians display a number of behaviors, such as phototaxis and thermotaxis, in response to external stimuli, and it has been shown that various molecules and neural pathways in the brain are involved in controlling these behaviors. However, due to the lack of combinatorial assay methods, it remains obscure whether planarians possess higher brain functions, including integration in the brain, in which multiple signals coming from outside are coordinated and used in determining behavioral strategies.

**Results:**

In the present study, we designed chemotaxis and thigmotaxis/kinesis tracking assays to measure several planarian behaviors in addition to those measured by phototaxis and thermotaxis assays previously established by our group, and used these tests to analyze planarian chemotactic and thigmotactic/kinetic behaviors. We found that headless planarian body fragments and planarians that had specifically lost neural activity following regeneration-dependent conditional gene knockdown (Readyknock) of synaptotagmin in the brain lost both chemotactic and thigmotactic behaviors, suggesting that neural activity in the brain is required for the planarian's chemotactic and thigmotactic behaviors. Furthermore, we compared the strength of phototaxis, chemotaxis, thigmotaxis/kinesis, and thermotaxis by presenting simultaneous binary stimuli to planarians. We found that planarians showed a clear order of predominance of these behaviors. For example, when planarians were simultaneously exposed to 400 lux of light and a chemoattractant, they showed chemoattractive behavior irrespective of the direction of the light source, although exposure to light of this intensity alone induces evasive behavior away from the light source. In contrast, when the light intensity was increased to 800 or 1600 lux and the same dose of chemoattractant was presented, planarian behaviors were gradually shifted to negative phototaxis rather than chemoattraction. These results suggest that planarians may be capable of selecting behavioral strategies via the integration of discrete brain functions when exposed to multiple stimuli.

**Conclusions:**

The planarian brain processes external signals received through the respective sensory neurons, thereby resulting in the production of appropriate behaviors. In addition, planarians can adjust behavioral features in response to stimulus conditions by integrating multiple external signals in the brain.

**Electronic supplementary material:**

The online version of this article (doi:10.1186/s40851-014-0010-z) contains supplementary material, which is available to authorized users.

## Introduction

As an animal survives under exposure to many kinds of stimuli, its nervous system detects sensory cues and converts this information into adaptive movement. For behaviors in response to a simple stimulus, sensory neurons sometimes communicate directly with motor neurons; however, when animals are exposed to more complex stimuli, integration of sensory information should be necessary to decide the appropriate behavior. Furthermore, integration of sensory information in this neural machinery is essential for choosing an animal's behavioral strategy based on the context and on the animal's memory, and such integration enables animals to refine their behaviors. Although some of the specific neuronal processing activities that encode neuronal activation into a behavioral response have been extensively studied, much remains to be understood about how to these processing activities decide an adaptive behavioral strategy under multiple environmental signals.

Planarians are free-living platyhelminths, and belong to an evolutionarily early group possessing a CNS that includes a brain with simple architecture, i.e., a bi-lobed brain composed of around 2.0 ~ 3.0 x 10^4^ neurons in a planarian of length about 8 mm [1-3]; and their brain consists of several functional and structural domains defined by the discrete expression of homeobox genes, with a surprisingly complex set of expressed genes, sophisticated neural networks, and neural modulators that are quite similar to those used by mammals [4]. In addition, planarians can sense a variety of environmental signals, and rapidly display distinct responsive behaviors depending on the type of signal, such as light or temperature [5-7], conveyed through sensory neurons projecting to their brain [1,2]. Despite the increasing knowledge that has been gained recently about the morphogenesis of the planarian brain and its robust regenerative ability [4,8], examination of the function of the planarian brain at the molecular level has just begun [6,7,9-11]. Research during the past two decades using molecular and cellular techniques has shown that the planarian brain is divided into several functional and structural domains that are composed of several neural subtypes, and that it uses many neurotransmitters and neuronal modulators, such as glutamate, dopamine, serotonin, GABA, acetylcholine, and neuropeptides, that are quite similar to those used by mammals [12-16]. These findings indicate that analysis of the planarian brain with its structurally simple, but nevertheless well-organized, brain may provide a unique opportunity as an emerging good new model system to elucidate molecular mechanisms underlying the basis of brain function. However, it has been difficult until now to clarify the mechanisms of planarian higher brain function, including learning and memory, because of the lack of knowledge about the neural processing pathway(s) in the brain regulating the behavior in response to a particular stimulus or to multiple stimuli.

Planarians display stereotypical behaviors in response to external stimuli, for example, they display phototaxis, chemotaxis, thermotaxis, and thigmotaxis [5]. Phototaxis and chemotaxis of planarians have been relatively well studied because of their association with morphologically well-characterized organs, namely the eyes and auricles, respectively [6,11,17]. The sensory organs of planarians are located in the head portion of the animal and send projections to the brain. The brain processes these signals and directs appropriate behavioral responses [6,11]. These findings clearly showed that planarian behavioral assays are useful for analyzing the CNS function. In this study, we focused on chemotaxis and thigmotaxis in addition to phototaxis and thermotaxis, and thereby assessed planarian behaviors that might reveal molecular and neural pathways in the brain involved in producing appropriate behavior in response to multiple signals.

## Materials and methods

### Animals

A clonal strain of planarian (*Dugesia japonica*), SSP, cultured at 23°C in tap water was used. Planarians were starved for at least one week prior to amputation, anesthetized by chilling on ice, and then cut. Planarians 7 mm in length were used for all experiments. All planarians were maintained and manipulated according to a protocol approved by the Animal Care and Use Committee of Kyoto University.

### Assay for planarian behaviors

**Figure 1:**
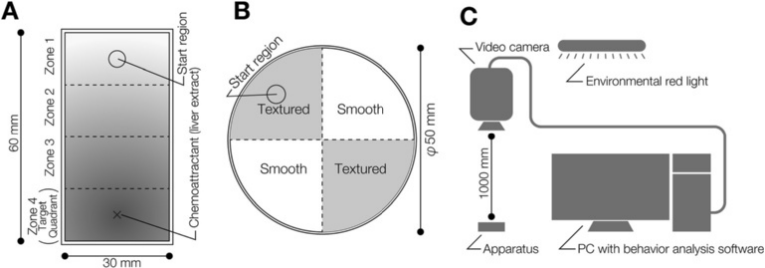
**Schematic drawings of the assay system for chemotaxis and thigmotaxis/kinesis. (A)** The assay chamber used to test chemotaxis. The cross indicates the peak of the chemoattractant gradient. The circle indicates the start region. Planarian behavior was quantified using the time spent in the target quadrant (Zone 4). **(B)** The assay plate used to test thigmotaxis/kinesis. The 2 opposing quadrants shaded gray indicate the textured regions. The white quadrants indicate smooth regions. The circle indicates the start region. Planarian behavior was quantified using the time spent in the two smooth regions. **(C)** Planarian behavior was recorded using a digital video camera and was analyzed using a computer and behavior analysis software.

A schematic representation of the chemotaxis assay system is shown in Figure 1A. Two ml of water containing 0.1% low melting point agarose at 23°C was placed in a 60 × 30 × 10 mm assay chamber. To obtain reproducible assay results, chicken liver extract prepared as follows was used as the chemoattractant: small chicken liver pieces were autoclaved (121°C, 20 min); the supernatant was obtained (chicken liver extract). The liver extract was divided into aliquots and frozen until use. Ten μl of the 100-fold diluted extract were placed in the center of the target quadrant (Zone 4) at one end of the assay chamber. A planarian was placed in the quadrant at the opposite end (Zone 1) of the chamber (Figure 1A).

A schematic representation of the thigmotaxis assay system is shown in Figure 1B. The surface of two opposing quadrants of a 50-mm-diameter plastic Petri dish were sanded (“textured”) using #12 sandpaper, as shown in Figure 1B. Planarians were placed on these textured regions of the assay plate that had been covered with 5 ml of autoclaved tap water at 23°C. The thermotaxis assay was performed as described previously [7].

For assays to examine the priorities among the four planarian behaviors studied here (chemotaxis, phototaxis, thigmotaxis, and thermotaxis), two behaviors were tested simultaneously as follows. A planarian was placed in the center of the 60 × 30 × 10 mm assay chamber described above. A preference index was calculated as follows: preference index = (number of planarians in one particular region – number of planarians in the opposite region)/(total number of planarians in the assay chamber).

To assay for external stimuli integration, a planarian was placed in the middle of a 60 × 10 × 3 mm chamber constructed from glass [6]. One ml of water containing 0.1% low melting point agarose was put in the container. Each planarian behavior was captured using a video recorder (Sony) placed above the container (Figure 1C) for the time indicated in the Results section. The trajectory of movement was analyzed with SMART software (Panlab) (Figure 1C) [6]. All behavioral experiments were performed in a dark room with only a red light of a wavelength that cannot be sensed by planarians (Figure 1C) [18]. Calculations based on the data obtained were performed using static ggplot2 package of R software [19].

### Statistical evaluation

Data were analyzed by determining the statistical significance of differences between test results as determined by Student's t test; *p* values greater than 0.05 were taken as not significant (ns).

### RNA interference

Double-stranded RNA (dsRNA) was prepared as described previously [20,21]. For Readyknock [11], dsRNA was injected into the posterior intestinal duct of planarians using a Drummond Scientific Nanoject injector (Broomall, PA, USA). At four hours after injection, planarians were amputated posterior to the auricles and the resulting regenerants were used for analysis at seven days of regeneration. Control animals were injected with dsRNA for green fluorescent protein (GFP), a gene that is not found in planarians.

### Whole-mount immunohistochemistry

Whole-mount immunostaining was performed as described previously [10]. Planarians were stained using the following dilutions of antibodies: 1/2000 anti-planarian synaptotagmin (anti-DjSYT) [22], 1:1000 anti-planarian arrestin [23], 1:2000 anti-G-protein β subunit (anti-DjGβ) [10], or 1:2000 anti-planarian tyramine beta-hydroxylase (anti-DjTBH) [24], in 10% goat serum in 0.1% Triton X-100-containing phosphate buffered saline (TPBS). After washing, the samples were incubated with fluorescently labeled goat secondary antibodies (Alexa488-labeled anti-mouse IgG(H + L) antibody and 10 μg/ml Hoechst 33342 (Life Technologies) in TPBS containing 10% goat serum overnight at 4°C. Fluorescence was detected with an FV10 confocal scanning microscope (Olympus) (10x/0.4 NA, or 60x/1.34 NA oil immersion objective lens). Images were processed with FV10-ASW (Olympus) and ImageJ software (NIH). All images were obtained using the same photography conditions to allow direct comparison between experimental animals and controls.

## Results

### Chemotactic behavior analysis in planarian

**Figure 2:**
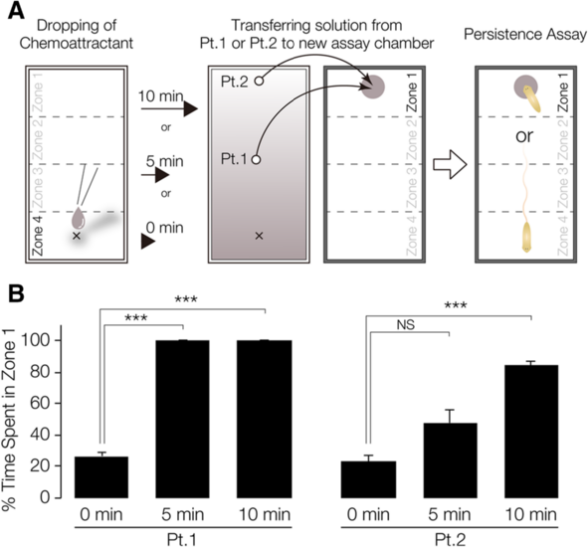
**Persistence assay for assessing the formation of a chemoattractant concentration gradient. (A)** Schematic illustration of the method of the persistence assay. Ten μl of chicken liver extract were placed in the center of one quadrant (Zone 4) of the assay chamber containing 2 ml of 0.1% low melting point agar solution. Zero, 5, or 10 min later, 3 μl of the agar solution was transferred from point 1 or point 2 to the center of a quadrant (Zone 1) of a fresh chamber, and then a planarian was placed in Zone 1. Planarians normally move around continuously after transfer to an assay chamber or culture dishes, and therefore the time spent in Zone 1 would normally gradually decrease. However, they remain in the initial region (Zone 1) if a sufficiently high concentration of chemoattractant is present there. **(B)** Time spent in Zone 1 during 1-min assay period. The chemoattractant diffused from Zone 4 to Zone 1 within 10 min after it was added.

To observe and quantify planarian chemical-sensing behavior, a tractable assay method for tracking chemotaxis behavior was developed. For this assay, we used liver-extract solution, the food used for culturing planarians in our laboratory, as chemoattractant (Figure 1A). We reasoned that if planarian recognized the chemoattractant and showed chemotaxis toward it, the chemoattractant should be present with a concentration gradient in the assay field. However, because we could not visualize or measure the concentration gradient of the chemoattractant(s) sufficient to induce the planarian chemotaxis, we instead used a bioassay we named the “persistence assay” (Figure 2A). The rationale for the “persistence assay” is that whereas planarians normally move in various directions and away from the original region (Zone 1) after being placed there, they would remain in the original region (Zone 1) if a concentration of chemoattractant sufficient for them to sense were present there. We preliminarily measured the chemoattractant's diffusion rate in the assay field to determine when we should start the analysis after adding the chemoattractant as follows. Ten μl of chicken liver extract as chemoattractant were placed in the center of a quadrant (Zone 4) of the assay chamber. After 0, 5, or 10 min, 3 μl of agar solution was transferred from point 1 or point 2 into the center of a quadrant (Zone 1) of a fresh assay chamber. Soon thereafter, a planarian was placed in Zone 1, and its behavior was observed. Figure 2B shows the time spent in Zone 1 during the 1-min assay period. Although at 0 min after adding the solution transferred from point 1, planarians moved away from Zone 1 and showed a low score of time spent there (percent of time ± SEM, 26.7 ± 2.9%), after 5 min planarians continuously stayed in Zone 1, and thus showed a higher score of time spent there (100 ± 0.0%) (Figure 2B). In contrast, planarians did not stay in Zone 1 even after 5 min (47.8 ± 8.31%), when the solution had been transferred there from point 2. However, after 10 min, they did stay in Zone 1 (84.3 ± 3.1%). In addition, we did not find any differences in locomotion among these planarians (data not shown). These results suggest that the chemoattractant reproducibly diffused throughout the entire assay chamber within 10 min after it was added, and that this bioassay using planarians is useful for directly and efficiently detecting the diffusion of a chemoattractant that induced planarian chemotaxis. Therefore, we decided to use the chemoattractant gradient field in the assay chamber 10 min after adding the chemoattractant to it in subsequent experiments.

**Figure 3:**
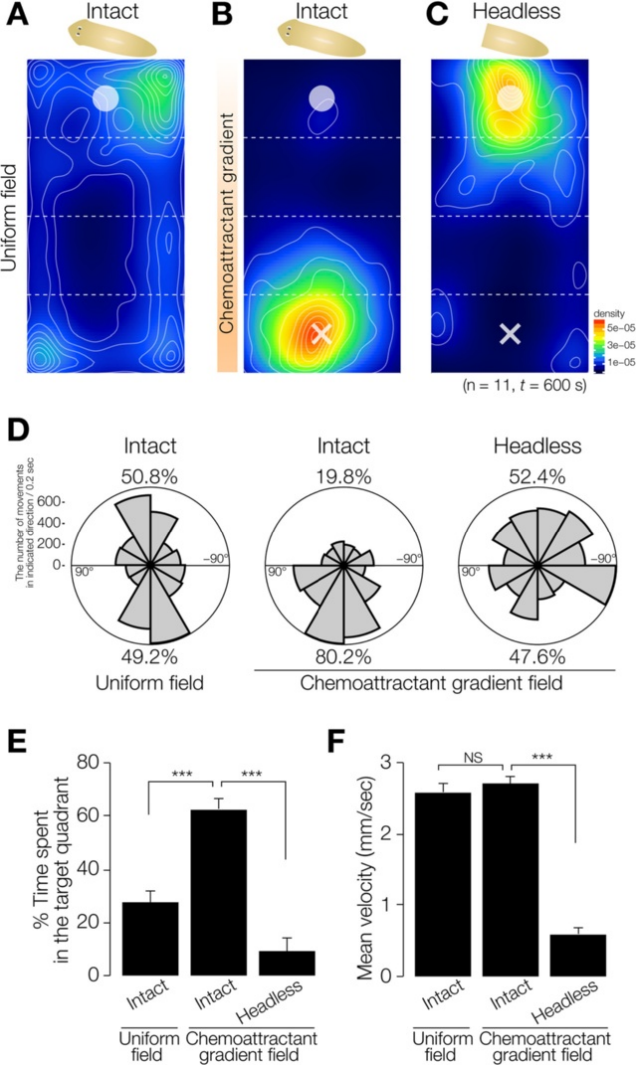
**Chemotaxis of intact and headless planarians. (A)** Heat map view with contour lines of the averaged behavior of 11 individually assayed intact animals in a uniform field. Planarian showed a preference for moving along the edge of a uniform chamber. **(B)** Heat map view with contour lines of chemotaxis of intact planarians in chemoattractant concentration gradient field. **(C)** Heat map view with contour lines of chemotaxis of headless planarians in chemoattractant concentration gradient field. Planarians showed a preference for moving along the edge of the uniform-field dish. In contrast, intact planarians moved to and stayed in the region with the highest concentration of chemoattractant in the chemoattractant-gradient field. Headless planarians did not show such chemotaxis. **(D)** Rose plots show orientation of movement of intact and headless planarians in Zones 2 and 3 in a uniform-field and chemoattractant gradient-field. The movement of intact planarians in a uniform-field, and that of headless animals in a chemoattractant gradient-field, showed no particular orientation, whereas the movement of intact planarians was biased toward being oriented toward the highest concentration of chemoattractant. **(E)** Time spent in the target quadrant (Zone 4) during assay of intact and headless planarians in the uniform-field and chemoattractant gradient-field is shown as mean ± SEM. **(F)** Mean velocity of intact and headless planarians during assay. ***, *p* < 0.005; NS, not significant; *t* = 600 sec; n = 11.

Next, we tested the behavior of intact planarians in a normal field (uniform-field assay chamber without chemoattractant). Figure 3A shows the averaged movements of 11 planarians together with a heat map for these movements (in which warm colors indicate locations where much time was spent, and cool colors those where little time was spent), and indicates that planarians tended to move near the edge of the field. In contrast, planarians placed at the start region indicated by an open circle in a chamber with chemoattractant showed a preference to move toward the region where the chemoattractant had been dropped (cross in Figure 3B). However, headless planarians did not move toward the chemoattractant, and instead showed random movements around the start region indicated by a white circle in Zone 1, indicating that the head is required for chemotaxis (Figure 3C). In order to investigate whether planarians orient their movement up a gradient of chemoattractant, we analyzed the overall orientation (angle) of their movement in Zones 2 and 3 (except for the start and target quadrants). Intact animals in a chemoattractant gradient field showed orientation biased toward the chemoattractant (80.2% of their movement was directed toward the chemoattractant), whereas planarians in a uniform field (49.2%) and headless planarians (47.6%) did not show linear movement directed toward the chemoattractant, and instead showed random movements (Figure 3D).

Next, the average time (during a 600-sec test interval) spent by the animals in the target quadrant region where the chemoattractant had been placed was measured to assess the ability of animals to recognize attractant chemicals and move to the region where those chemicals were concentrated. Intact animals spent a large fraction of their time in the target zone after reaching it (62.7 ± 4.1%) (Figure 3E). In contrast, headless animals showed a much lower thermotaxis score (8.9 ± 5.1%) (Figure 3E). There was no difference in the speed of movement of planarians in the chemoattractant-gradient field (2.69 ± 0.12 mm/sec) and uniform field (2.58 ± 0.11 mm/sec), indicating that the difference of chemotaxis score between animals on a chemoattractant-gradient, and thus the difference of planarian movement between a uniform-field chamber and a plate containing chemoattractant, was not the result of acceleration of the locomotor activity by the chemoattractant (Figure 3F). These results indicate that this assay method is useful for quantitatively evaluating planarian chemotactic behavior, and that planarian chemotaxis is dependent on the head. Although decapitation inhibited planarians’ movement, and they frequently stopped, resulting slower value of velocity (0.60 ± 0.07 mm/sec) (Figure 3F), the 600-sec assay time was thought to be sufficiently long for planarians to move to the target quadrant, suggesting that the headless planarians may have lost chemotaxis rather than that they spent less time in the target quadrant due to slowing of their movement.

### Analysis of the brain neurons involved in chemotaxis with Readyknock of *synaptotagmin*

**Figure 4:**
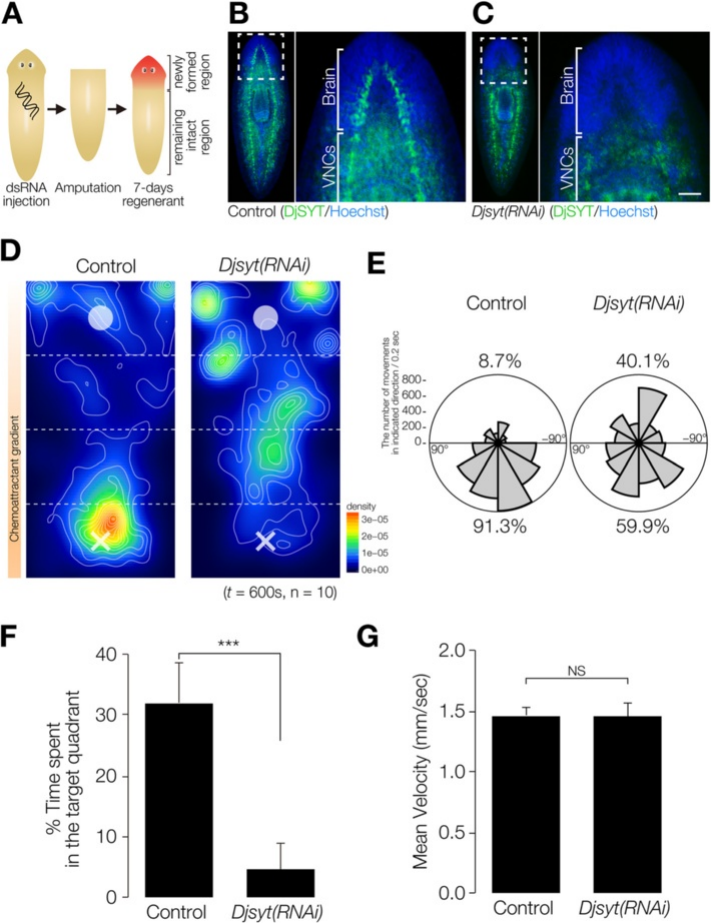
**Chemotaxis of planarians that had lost of brain neural activity by Readyknock of synaptotagmin gene. (A)** Schematic illustration of experimental design of Readyknock of synaptotagmin gene. After injection of double-stranded RNA of *Djsyt*, planarians were amputated, and then allowed to regenerate their heads for 7 days. Red-colored portion indicates newly regenerated head. **(B, C)** Control and Readyknock of *Djsyt*. Immunohistochemical detection of DjSYT protein, shown in green in control **(B)** and in Readyknock **(C)** animals 7 days after decapitation. Samples were stained with Hoechst 33342 (for nuclei, shown in blue) to visualize planarian tissues, including brain. The dashed boxes indicate the border between the newly formed head region magnified in the right panels. Readyknock using *Djsyt(RNAi)* treatment caused severe reduction of the level of DjSYT protein, although strong signals of the DjSYT protein were still detected in the pre-existing ventral nerve cords (VNCs) in the trunk region. Bar, 150 μm. **(D)** Heat map view with contour lines of chemotaxis of control and *Djsyt(RNAi)* planarians in chemoattractant concentration gradient field. *Djsyt(RNAi)* planarians showed random movement, whereas control planarians moved to and stayed in the region having the highest concentration of chemoattractant in the chemoattractant-gradient field. **(E)** Rose plots show orientation of movement of control and *Djsyt(RNAi)* planarians in Zones 2 and 3 in chemoattractant gradient-field. The movement of control planarians was biased toward being orientated toward the highest concentration of chemoattractant, whereas *Djsyt(RNAi)* animals in the chemoattractant gradient-field showed no particular orientation of movement. **(F)** Time spent in the target quadrant (Zone 4) during assay of control and *Djsyt(RNAi)* planarians in the chemoattractant gradient-field is shown as mean ± SEM. **(G)** Mean velocity mean of control and *Djsyt(RNAi)* planarians during assay. ***, *p* < 0.005; NS, not significant; *t* = 600 sec; n = 10.

The above data showed that headless planarian showed slower movement, and therefore we next analyzed various brain neurons to test whether the brain was required for chemotaxis. In order to perturb the activity of brain neurons, we performed regeneration-dependent conditional gene knockdown (Readyknock), which knocks down protein expression more severely in the differentiating cells in the regeneration blastema than in the pre-existing terminally differentiated cells [7,11], using dsRNA of the gene encoding the planarian *synaptotagmin* (*Djsyt*), which is involved in synaptic transmission [22] (Figure 4A). Immunohistochemical analysis revealed the presence of DjSYT in the axons in the brain and VNCs (Figure 4B). Next, Readyknock using *Djsyt(RNAi)* treatment caused severe reduction of the level of DjSYT protein only within the newly formed brain in the head seven days after amputation, whereas strong signals of the DjSYT protein were detected in the pre-existing VNCs in the trunk region (Figure 4C). Previous reports indicated that *Djsyt(RNAi)* planarians cannot distinguish the direction of light or a thermal-gradient, and moved randomly when they were exposed to light or temperature stimuli [7,11]. To investigate the brain functions involved in chemotaxis, the chemotactic behavioral assay was carried out after Readyknock with *Djsyt(RNAi)* and revealed that *Djsyt(RNAi)* planarians did not preferentially move toward a chemoattractant, although control animals did (Figure 4D). In order to investigate whether a lack of the activity of the brain neurons would impair the linear movement toward chemoattractant that was seen in control planarians, we analyzed the overall direction (angle) of movement in Zones 2, and 3 (except in the start and target quadrants). In control animals, almost all movements were directed toward the chemoattractant, whereas *Djsyt(RNAi)* planarians were clearly less able to orient their movement in the correct direction toward the chemoattractant, and instead showed random movements (Figure 4E). When we calculated the fraction of movements directed toward the chemoattractant by dividing the overall direction of movement into two directions—the angle toward chemoattractant (+180°) and that in the opposite direction (–180°)—in the control animals, 91.3% of their movement was directed toward the chemoattractant, whereas *Djsyt(RNAi)* animals showed 40.1% of their movement directed away from the chemoattractant (Figure 4E). Quantitative analysis of time spent in the target quadrant (Zone 4), where the concentration of chemoattractant was highest, clearly demonstrated that the loss of DjSYT in the brain inhibited planarian chemotaxis (Figure 4F), without causing any defect in locomotor activity (Figure 4G). These results strongly suggest that neural activity in the brain is required for planarian chemotactic behavior, and that the chemotaxis assay system is useful for analyzing the function of the planarian brain and nervous-system-related genes.

### Thigmotactic/kinetic behavior analysis in planarians

**Figure 5:**
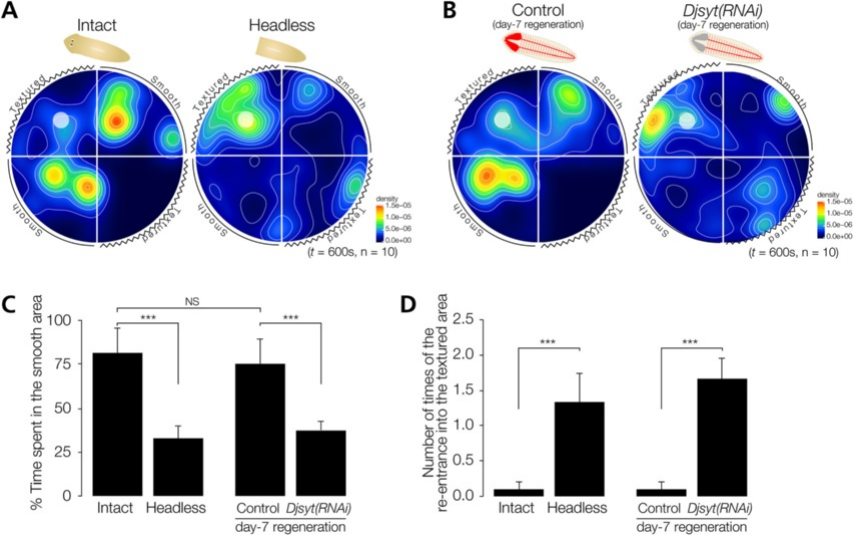
**Planarian thigmotaxis/kinesis. (A)** Heat map view with contour lines of thigmotaxis/kinesis of intact and headless planarians in thigmotaxis/kinesis assay field. Intact planarians tended to move to the smooth-surface region after starting from the textured surface region indicated by the white circle. In contrast, headless planarians continued to move around in the assay field independent of whether the bottom surface was smooth or textured. **(B)** Heat map view with contour lines of thigmotaxis/kinesis of control and Readyknock-treated planarians of *Djsyt* in the thigmotaxis/kinesis assay field. *Djsyt(RNAi)* planarian showed random movement, like headless planarians, whereas control planarians moved to and stayed in the smooth-surface region. **(C)** Time spent in the smooth region during assay of intact, headless, control, and *Djsyt(RNAi)* planarians in the thigmotaxis/kinesis assay field is shown as mean ± SEM. **(D)** The number of re-entries into a textured region from a smooth-surface region of intact, headless, control, and *Djsyt(RNAi)* planarians during the assay. ***, *p* < 0.005; NS, not significant; *t* = 600 sec; n = 10.

Planarians show reactions through mechanical-tactile sensing to such stimuli as water flow, touch, and contact with objects [5,25]. To study such sensing, we next established a thigmotaxis/kinesis assay (Figure 1B). Figure 5A shows the averaged movements of 10 planarians together with a heat map of these movements, and indicates that planarians placed at a start region with a textured surface region showed a preference to move away from the textured surface region and move to a region with a smooth surface, and then stopped on the smooth region (Figure 5A). Note that intact animals that started from a textured surface region little spent time in the textured surface region of the assay plate opposite to that of the start region. In contrast, headless planarians showed random movements, and stopped without regard to the start-site condition, indicating that the head is required for thigmotaxis/kinesis (Figure 5A). Next, to investigate brain functions involved in thigmotaxis/kinesis, we performed a thigmotaxis/kinesis behavioral assay after Readyknock with *Djsyt(RNAi)*. The results revealed that *Djsyt(RNAi)* planarians did not preferentially move to the smooth-surface region, although control animals showed normal thigmotactic/kinetic behavior (Figure 5B). In order to better analyze the data, we quantified behaviors by calculating the average time spent by the animals in the smooth-surface region during a 600-sec test period to assess the ability of animals to recognize the physical properties of the surface and to move to a smooth-surface region, and plotted the results graphically (Figure 5C). These analyses clearly indicate that intact animals spend a large fraction of their time in the target zone after reaching it (81.4 ± 14.2%) (Figure 5C), whereas headless animals show a much lower thigmotaxis/kinesis score (32.8 ± 7.3%) (Figure 5C). Similarly, quantification of the time spent in the smooth-surface region by *Djsyt(RNAi)* planarians clearly showed that although control RNAi animals showed normal thigmotactic/kinetic behavior (75.3 ± 14.3%) (Figure 5C), *Djsyt(RNAi)* animals moved randomly and stopped in a random manner, like headless planarians (37.5 ± 5.0%) (Figure 5C). This finding is consistent with the findings in our chemotaxis, phototaxis, and thermotaxis assays (Figure 4) [7,11].

Next, comparison of intact and control RNAi animals at seven days of regeneration revealed that the average time they spent in the smooth-surface region was nearly the same, indicating that the thigmotactic/kinetic behavior in planarian was fully recovered within seven days after amputation (Figure 5C). Furthermore, when we measured the number of times of that a planarian re-entered the textured-surface region once it had exited from that region (Figure 5D), the data showed that intact and control RNAi animals rarely re-entered the textured-surface region (average number of times ± SEM; number of individuals that re-entered the textured-surface region, 0.1 ± 0.1, 1/10; 0.1 ± 0.1, 1/10), whereas headless and *Djsyt(RNAi)* planarians frequently re-entered the textured-surface region (1.3 ± 0.4, 7/9; 1.7 ± 0.3, 8/9), indicating that planarians avoid textured surfaces, and that this behavior may require brain neural activity (Figure 5D). These results suggest that the thigmotaxis/kinesis assay system is useful for analyzing the function of the planarian brain and nervous-system-related genes, and that neural activity in the brain is required for several planarian behaviors, including chemotaxis, thigmotaxis/kinesis, phototaxis, and thermotaxis [7,11].

### Prioritization among behaviors

Next, by combining assays of different behaviors, namely chemotaxis, phototaxis, thermotaxis, and thigmotaxis/kinesis, we examined the ability of planarians to integrate various stimuli. To determine the order of predominance of these planarian behaviors under specific conditions in this study using constant strengths of stimuli, at first we performed combinatorial assays in which two distinct stimuli were presented simultaneously to planarians. For these assays, a planarian was placed in the center position of a 60 × 30 × 10 mm assay chamber, and was given different stimuli from the two different ends, and then the number of planarians at a given position was measured after 600 sec.

**Figure 6:**
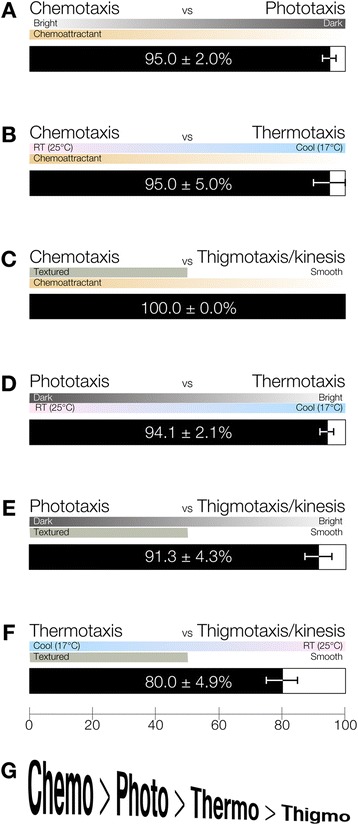
**Binary competitive behavior analyses. (A-F)** The order of predominance of the four tested behaviors. Chemotaxis vs Phototaxis **(A)**, Chemotaxis vs Thermotaxis **(B)**, Chemotaxis vs Thigmotaxis/kinesis **(C)**, Phototaxis vs Thermotaxis **(D)**, Phototaxis vs Thigmotaxis/kinesis **(E)**, and Thermotaxis vs Thigmotaxis/kinesis **(F)**. *n* = 20; t = 300 sec. **(G)** Under the conditions of this study, planarians gave first priority to chemotaxis, and lowest priority to thigmotaxis/kinesis.

When we compared behaviors in this combinatorial assay using planarians presented with both chemoattractant and 400 lux of light, planarians preferred to move toward the chemoattractant rather than escaping from the light, even though they received strong enough light (400 lux) to induce phototaxis in a single-stimulus assay (Figure 6A) [6]. The preference index for chemotaxis (95.0 ± 2.0%) clearly indicated that planarians predominantly showed chemotaxis rather phototaxis behavior (Figure 6A). Moreover, planarian chemotaxis was dominant over thermotaxis and thigmotaxis/kinesis (Figure 6B,C). Next, when phototaxis was compared to thermotaxis and thigmotaxis/kinesis, phototaxis was predominant over thermotaxis and thigmotaxis/kinesis (Figure 6D, E). Finally, when thermotaxis and thigmotaxis/kinesis were compared, the preference index of thermotaxis (80.0 ± 4.9%) was higher than that of thigmotaxis/kinesis (Figure 6F). The results of our analyses using these combinatory assay systems (chemotaxis, phototaxis, thermotaxis, and thigmotaxis/kinesis) revealed that one behavior tends to predominate when planarians receive two different stimuli (Figure 6A-F). In these combinatory experiments, planarians gave top priority to a chemical stimulus and second-highest priority to a light stimulus, and gave the lowest priority to a mechanical stimulus (Figure 6G). These data suggest that planarians may have the ability to integrate various different external kinds of information in the brain.

### Establishment of a behavioral integration assay method to analyze brain function using two distinct external stimuli

**Figure 7:**
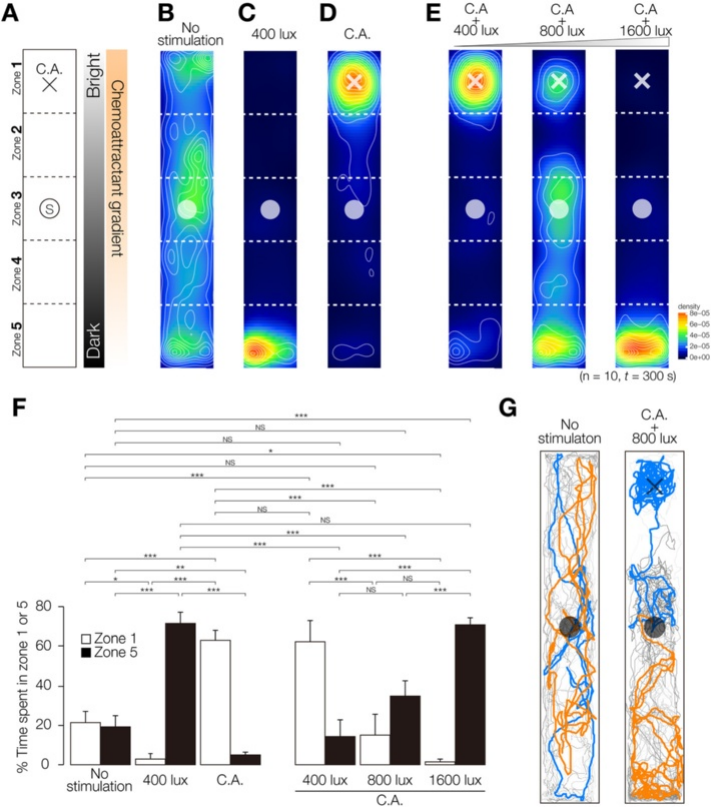
**Integration assay in planarian. (A)** Schematic illustration of the assay method used to investigate the integration of external stimuli is shown. A container made using glass as described in Materials and Methods was used for the experiments. A planarian was placed in Zone 3 (indicated by a circle). Three microliters of chemoattractant (C. A.) was dropped into Zone 1 (indicated by a cross). The chamber was illuminated from Zone 1 in the direction of Zone 5. **(B)** The behaviors of 10 planarians without any stimulation were analyzed independently in the chamber; they showed random movements. **(C)** The chamber was illuminated with 400 lux of light from the side of Zone 1. The planarians immediately moved away from the light toward Zone 5. **(D)** Chemoattractant (C. A.) was dropped into Zone 1. Planarians moved to Zone 1, where the chemoattractant was most highly concentrated. **(E)** Chemoattractant was dropped into Zone 1, which was then exposed to 400, 800, or 1600 lux of light. **(F)** Time spent in Zone 1 (white columns) and Zone 5 (black columns). **(G)** Trajectories of 10 planarians starting from the circle in Zone 3 without any stimulation (left) and with exposure to both 800 lux of light and chemoattractant simultaneously. Two representative trajectories were colored in orange and blue. Although no significant difference was seen in time spent in Zone 1 or 5 between no stimulation and multiple stimulation of 800 lux light plus chemoattractant, each control planarian moved extensively in the container, whereas each planarian given 800 lux of light and chemoattractant moved toward the chemoattractant or moved away from the light toward the dark region.

It is thought that animals integrate multiple signals, and show appropriate behaviors after integrating their responses. Next, to investigate in more detail whether planarians prioritize different stimuli, and whether the order of predominance of behaviors in planarian is absolute, we employed an integrative assay that we developed to perform combinatory assays using two distinct stimuli. Our combinatory assay system using two distinct stimuli, light and chemoattractant, is illustrated in Figure 7A. The 60 × 10 × 3 mm assay chamber was divided into five zones to enable quantitative measurement of the planarian behaviors. When a planarian was placed into the center of the chamber (Zone 3), it moved randomly (Figure 7B). The heat map of planarian movement showing the time spent in each zone did not indicate any particular tendency of movement in this control condition, which was consistent with the above data shown in Figure 3A. When we assayed planarian phototaxis and chemotaxis using this chamber, planarians moved away from the 400-lux light source (Figure 7C), as previously described [6], and planarians moved toward Zone 1, where the chemoattractant (C.A.) had been added (Figure 7D), as seen in Figures 3 and 4. In addition, when planarians were exposed to 400 lux of light and chemoattractant simultaneously, they moved toward the region of the chemoattractant source (Zone 1) (Figure 7E). This result was also consistent with the above results (Figure 6A). However, by exposing planarians to different intensities (800 or 1600 lux) of light and exposing them to a constant dose of chemoattractant at the same time, it was found that planarians changed their preference and moved toward the dark side of the chamber, depending on the level of the stimuli (Figure 7E). These results suggest that planarians can change their behavioral features in response to a stimulus to which they are repeatedly exposed, and that planarian behavior can be manipulated. Figure 7F shows the time spent in the darkest zone (Zone 5) and in the chemoattractant-source zone (Zone 1), and this graph clearly indicates that we successfully manipulated the outcome of the integration process by changing the signal strength. When planarians were exposed to 800 lux of light and chemoattractant simultaneously, they seemed to move in a random way, as seen in control planarians without stimulation (Figure 7B). Consistently, there was no significant difference in spent time in Zone 1 or 5 between planarians with no stimulation and combined exposure to 800 lux of light and chemoattractant (Figure 7F). However, careful analysis of their trajectories revealed that each control planarian moved extensively in the assay chamber, whereas each planarian exposed to 800 lux of light and chemoattractant moved toward the chemoattractant or moved toward the dark region (Figure 7G). This suggests that planarians might decide upon a behavior strategy from among several possible candidate strategies, and they may select different strategies depending on the individual. These results suggest that planarians may decide their behavioral strategies via their brain function when exposed to multiple stimuli.

## Discussion

### Planarian chemotaxis and thigmotaxis/kinesis

Chemosensing in addition to visual sensing and appropriate responses to various stimuli in order to find food, to escape from predators, and to communicate with other individuals, are among the most important primary functions of organisms. Planarian food-finding behavior is thought to be completely dependent on chemosensing [26], and the auricles and head margin are thought to be an important organ of chemosensing [27], although no chemosensory receptors or chemosensory neurons have been identified. Here, we established a new, tractable behavioral analysis system for chemotaxis to a food attractant, and used it to quantify the movements of planarians exposed to food as a chemoattractant. This behavioral assay system combined with RNAi may be a powerful tool for finding genes involved in chemotaxis, genes important in processing neurons in the brain, genes encoding chemosensory receptors, and other genes important in chemosensory neurons.

In this study, to evaluate the strength of a chemoattractant gradient that could be established by diffusion through a low-concentration agar gel, we made successive transfers of the chemoattractant solution, thereby successively diluting it, and directly examined whether the transferred solution was able to induce planarian chemotaxis (Figure 2). This bioassay may be useful for studying chemotaxis induced by substances for which we have no way of measuring the concentration gradient directly. When placed in this reproducible chemoattractant-gradient field, planarians spent the most time in the region containing the highest concentration of the chemoattractant (Figure 3B). Furthermore, the direction of the planarians’ movement clearly showed a tendency to move toward the region with the highest concentration of the chemoattractant (Figure 3D). These results clearly suggest that the planarian may recognize a concentration gradient of chemicals: they moved toward the higher concentration of the chemoattractant without showing increased kinesis.

In contrast, unfortunately we have not been able to prepare a texture-gradient field, and therefore we were unable to investigate how planarians recognize the difference between textured and smooth surfaces to reach a region with a preferred surface. In addition, we are unable to distinguish whether the thigmotaxis/kinesis we observed here was positive or negative (preference for a smooth surface or evasion of a textured surface), although the planarians showed a clear tendency to move to and stay for a long time in the smooth surface region (Figure 5). We speculate that planarians can attach more strongly to smooth surfaces with wider adhesion areas than to textured surfaces, and that this behavior may benefit planarians by enabling them to find safer and more stable surfaces. Consistently, in nature, planarians can usually be found under small stones or leaves, where they may avoid light, strong water flow, and predators. Moreover, we found that this feature of planarians could be utilized to arbitrarily restrict the regions where planarians moved in a field to regions without vertical walls (Additional file 1: Figure S1).

### Planarian behaviors require neural activity of the brain

The robust regenerative ability of planarians makes them very useful for analyzing the phenotypes of amputated body fragments, such as headless animals, in order to investigate the organs regulating behaviors, since these fragments do not die. Head-amputated planarians completely lost both chemotaxis and thigmotaxis (Figures 3C, and 5A), suggesting that the head is required for both of these planarian behaviors. Furthermore, the planarian's strong regenerative ability enabled us to perform region-conditional gene knockdown by the procedure called Readyknock [11], and the resultant specific loss of neural activity in the brain by Readyknock of the synaptotagmin gene caused complete loss of both chemotaxis and thigmotaxis. These results suggest that neural activity in the brain is required for the sensing of chemical and mechanical stimuli, and/or for processing in the brain of the signals received through chemo- and thigmosensory neurons.

Note that decapitation caused a decrease of mean velocity of locomotion (Figure 3F), although the duration of the assay (600 sec) was sufficient for planarians to reach target regions from the start point in the assay chamber, and for us to judge their chemotactic behavior. Because of this decrease of mean velocity we cannot completely exclude the possibility that the decapitation-induced decrease of velocity led to the reduction of the chemotactic and thigmotactic scores. However, loss of synaptotagmin gene function in the brain did not affect their velocity of locomotion (Figure 4G), but did cause loss of these behaviors, suggesting that planarian chemotaxis and thigmotaxis are dependent on the brain in their head, and that our chemotaxis and thigmotaxis assay systems are useful for analyzing various functions of the planarian brain and of nervous-system-related genes. Previously, we reported that neural activity in the brain is required for both phototaxis and thermotaxis [7,11,28]. Our results in this study together with those previous results strongly suggest that planarian behaviors responsive to external stimuli are controlled by the brain.

### Panel of behavioral assays may provide means to unravel neural networks of higher brain function in planarian

Planarian higher brain function was first described in the 1950s, when Thompson and McConnell used classical conditioning experiments using light and electrical shock to show planarian's ability to learn and remember [29]. However, as this phenomenon has not been verified by other groups, McConnell's studies of planarian learning remain controversial [30,31].

The unique features of planarians’ higher brain functions have provided many insights into brain function in metazoans, and studies on planarian classical conditioning have attracted many researchers [32,33]. However, the neural pathways of planarian higher brain functions, including integration and learning, have not yet been unraveled, and even many of the original observations made decades ago have not been verified yet at the neuronal level. Moreover, it is difficult to analyze the neural mechanisms underlying higher brain function in planarians using the originally reported classical conditioning assay system utilizing light and electrical shock, because electrical stimulation might affect muscles directly. Previously, assay systems for planarian phototaxis and thermotaxis have been reported, and a number of genes and neuronal subtypes involved in sensing external stimuli and related characteristic behaviors have been identified [6,7,28]. Here we took another step forward by developing quantitative methods for chemotaxis and thigmotaxis/kinesis that enabled us to investigate the mechanisms underlying information processing and integration in the planarian brain. In order to acquire information about planarian behaviors in response to multiple simultaneous stimuli, we performed a binary competitive assay in which a planarian was given two distinct stimuli simultaneously under specific conditions in combinatory experiments. The results showed that planarians displayed an order of priority among their responses to these different stimuli (Figure 6), suggesting that the planarian brain has the ability to integrate signals coming from outside to decide appropriate planarian behaviors. Furthermore, we showed that planarian movements vary according to the stimulus level (Figure 7E), although we did not find any light-intensity dependence of planarian phototaxis (data not shown). These results suggest that planarians do not show a simple, direct response to a stimulus, but rather integrate external stimuli and then behave suitably in response to the overall conditions.

### Neural networks predicted to be involved in controlling decision-making

**Figure 8:**
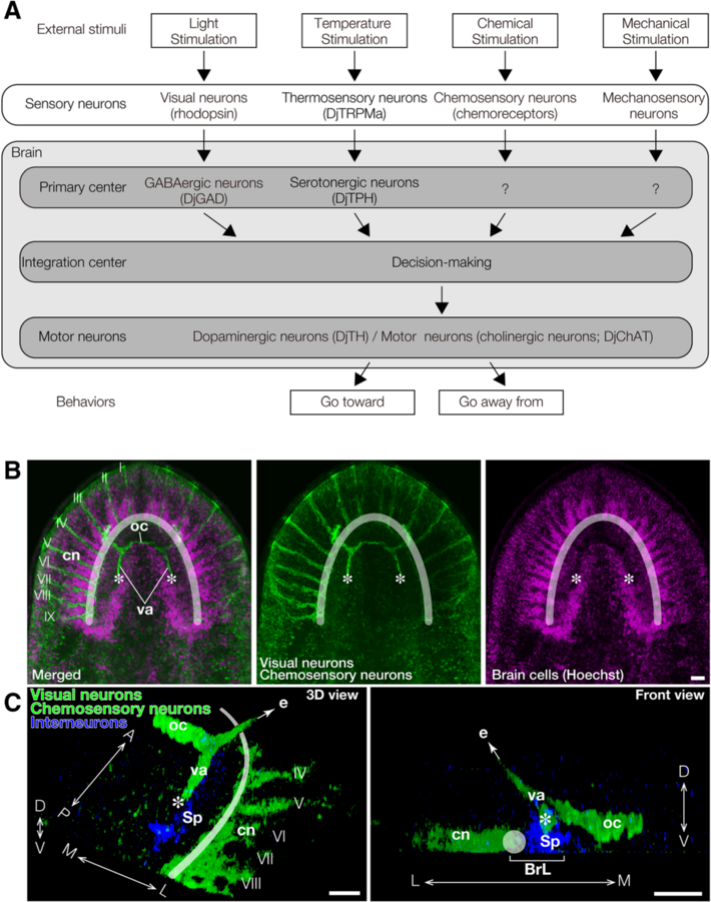
**Neural networks controlling planarian behaviors. (A)** Planarians receive many kinds of signals from the outside, through independent types of sensory neurons such as rhodopsin-expressing visual neurons, DjTRPMa-expressing thermosensory neurons, and chemoreceptor-expressing chemosensory neurons, and these signals are processed by neurons in the brain, such as GABAergic neurons and serotonergic neurons. Thereafter, the various signals are integrated via certain neural networks in the brain to decide a planarian's behavioral strategy. Subsequently, the planarian behaves suitably in response to its conditions by controlling its motor neurons. **(B, C)** Different projections of visual neurons visualized by immunohistochemical staining with anti-arrestin antibody and chemosensory neurons visualized by staining with anti-DjGβ. **(B)** Axons of visual neurons form the optic chiasm and project to the medial region of the brain indicated by asterisks. Chemosensory neurons are located in nine lateral brain branches whose dendrites elongate toward the outer region of the head (I - IX) and whose axons project to the lateral region of the brain indicated by a diaphanous white arc. **(C)** 3D view (left panel) and front view (right panel) of visual neurons (green), chemosensory neurons (green), and interneurons in the spongy region of the brain (blue). Axons of visual neurons project to the dorso-medial region of the brain indicated by asterisks, whereas axons of chemosensory neurons project to the ventro-lateral region of brain indicated by a diaphanous white arc or circle. BrL, brain lobe; e, eye; cn, chemosensory neuron; oc, optic chiasm; Sp, spongy region; va, visual axons; A, anterior; P, posterior; M, medial; L, lateral; D, dorsal, V, ventral. Bars, 200 μm.

Figure 8 shows all the planarian neural circuits predicted from this and previous studies. Several receptors for light and chemical stimuli were previously identified [23,34,35]. Regarding brain interneurons, RNAi studies of planarian glutamic acid decarboxylase (DjGAD) demonstrated the involvement of GABAergic neurons in phototaxis [28]. We found recently that planarian transient receptor potential ion channel melastatin family a (DjTRPMa*)* may be involved in sensing temperature, and that brain-specific *DjTPH(RNAi)* caused loss of thermotaxis, suggesting that serotonergic neurons in the brain are required for processing of thermosensing signals to produce thermotaxis [7]. To produce a behavior in response to a stimulus, planarian locomotion is coordinated by dopaminergic neurons [36], and motor function of body wall muscles is regulated by cholinergic neurons [37] (Figure 8A).

It seems unlikely that individual neurotransmitters are involved in only single neural circuits or behaviors [24,28,37]; rather, neurons expressing a particular neurotransmitter may have different roles according to the particular cell or position in the brain, and may accordingly construct more complex neural networks. By accumulating further data from the kinds of experiments reported here and further refining the assay systems, we should be able to unravel the neural networks or systems involved in the higher brain function in planarian. Here we found that planarian behaviors in response to stimuli could be changed flexibly according to the level of stimulation. Axons of the sensory neurons in planarian, such as the axons of the optic neurons and chemosensory neurons, project to different regions in the brain (Figure 8B,C). For example, visual neurons project to the dorso-medial region (visual center) in the brain, whereas axons of chemosensory neurons in the lateral branch neurons project to the ventro-lateral region of the brain, as visualized by immunostaining with specific antibodies (Figure 8B,C), consistent with previous results obtained using fluorescent dye tracing [2]. It has been suggested that external stimuli sensed by such organs are integrated in some region(s) of the brain, finally resulting in planarians’ behaviors. Ultimately, the information acquired by sensory neurons might be accumulated inside the brain, and then processed and integrated to transduce the signals into the activity of motor neurons (Figure 8).

## Conclusions

The chemotaxis and thigmotaxis/kinesis assay methods established here are useful for quantitatively analyzing planarian chemotactic and thigmotactic/kinetic behaviors. Headless planarian body fragments and planarians treated by Readyknock of synaptotagmin in the brain lost both chemotactic and thigmotactic behaviors, suggesting that brain neural activity is required for the chemotactic and the thigmotactic behavior in planarian. When we tested the priority among four planarian behaviors (phototaxis, chemotaxis, thigmotaxis/kinesis, and thermotaxis) by presenting the respective stimuli to planarians simultaneously as binary stimuli, the results revealed that planarians showed a clear order of predominance of these behaviors, with chemotaxis being the strongest stimulus under our conditions. We also found that planarians showed predominance of their behavioral response to either of two simultaneously presented stimuli depending on the strengths of the two stimuli in a competitive binary stimuli assay using light and chemoattractant. Taken together, our results support the notion that external stimuli sensed by respective sensory organs are integrated in the brain and determine planarian behaviors.

## Additional file

Additional file 1: Figure S1. Planarian behaviors in arbitrary smooth surface region. **(A)** Schematic drawing of textured dish for thigmotaxis/kinesis assay. Textured regions are indicated by gray color. The other region (colored white) indicates a smooth region. **(B)** Heat map view of planarians’ movement. Planarians only moved on the smooth surface region, although planarians usually tend to move near the edge of a dish as a default behavior.

## Abbreviations

CNS: Central nervous system
VNCs: Ventral nerve cords
RNAi: RNA interference
Readyknock: Regeneration-dependent conditional gene knockdown
CA: chemoattractant
DjGβ: G-protein β subunit
DjTBH: *Dugesia japonica* tyramine beta-hydroxylase
Djsyt: *Dugesia japonica* synaptotagmin
DjTRPMa: *Dugesia japonica* transient receptor potential ion channel melastatin family a
DjTPH: *Dugesia japonica* tryptophan hydroxylase
DjGAD: Glutamic acid decarboxylase; gamma-aminobutyric acid
DjTH: *Dugesia japonica* tyrosine hydroxylase
DjChAT: *Dugesia japonica* choline acetyltransferase
oc: optic chiasm
cn: Chemosensory neuron
SEM: Standard error of mean

## References

[1] Agata K, Soejima Y, Kato K, Kobayashi C, Umesono Y, Watanabe K (1998). Structure of the planarian central nervous system (CNS) revealed by neuronal cell markers. Zool Sci.

[2] Okamoto K, Takeuchi K, Agata K (2005). Neural projections in planarian brain revealed by fluorescent dye tracing. Zool Sci.

[3] Takeda H, Nishimura K, Agata K (2009). Planarians maintain a constant ratio of different cell types during changes in body size by using the stem cell system. Zool Sci.

[4] Umesono Y, Tasaki J, Nishimura K, Inoue T, Agata K (2011). Regeneration in an evolutionarily primitive brain–the planarian Dugesia japonica model. Eur J Neurosci.

[5] Pearl R (1903). The movements and reactions of fresh-water planarians: A study in animal behaviour. Q J Microsc Sci.

[6] Inoue T, Kumamoto H, Okamoto K, Umesono Y, Sakai M, Sanchez Alvarado A (2004). Morphological and functional recovery of the planarian photosensing system during head regeneration. Zool Sci.

[7] Inoue T, Yamashita T, Agata K (2014). Thermosensory signaling by TRPM is processed by brain serotonergic neurons to produce planarian thermotaxis. J Neurosci.

[8] Cebria F (2007). Regenerating the central nervous system: how easy for planarians!. Dev Genes Evol.

[9] Fusaoka E, Inoue T, Mineta K, Agata K, Takeuchi K (2006). Structure and function of primitive immunoglobulin superfamily neural cell adhesion molecules: a lesson from studies on planarian. Genes Cells.

[10] Inoue T, Hayashi T, Takechi K, Agata K (2007). Clathrin-mediated endocytic signals are required for the regeneration of, as well as homeostasis in, the planarian CNS. Development.

[11] Takano T, Pulvers JN, Inoue T, Tarui H, Sakamoto H, Agata K (2007). Regeneration-dependent conditional gene knockdown (Readyknock) in planarian: demonstration of requirement for Djsnap-25 expression in the brain for negative phototactic behavior. Dev Growth Differ.

[12] Umesono Y, Watanabe K, Agata K (1997). A planarian orthopedia homolog is specifically expressed in the branch region of both the mature and regenerating brain. Dev Growth Differ.

[13] Umesono Y, Watanabe K, Agata K (1999). Distinct structural domains in the planarian brain defined by the expression of evolutionarily conserved homeobox genes. Dev Genes Evol.

[14] Cebria F, Kudome T, Nakazawa M, Mineta K, Ikeo K, Gojobori T (2002). The expression of neural-specific genes reveals the structural and molecular complexity of the planarian central nervous system. Mech Dev.

[15] Cebria F, Nakazawa M, Mineta K, Ikeo K, Gojobori T, Agata K (2002). Dissecting planarian central nervous system regeneration by the expression of neural-specific genes. Dev Growth Differ.

[16] Nakazawa M, Cebria F, Mineta K, Ikeo K, Agata K, Gojobori T (2003). Search for the evolutionary origin of a brain: planarian brain characterized by microarray. Mol Biol Evol.

[17] MacRae EK (1964). Observations on the fine structure of photoreceptor cells in the planarian *Dugesia tigrina*. J Ultrastruct Res.

[18] Azuma K, Iwasaki N, Ohtsu K (1999). Absorption spectra of planarian visual pigments and two states of the metarhodopsin intermediates. Photochem Photobiol.

[19] Wickham H (2009). ggplot2: Elegant Graphics for Data Analysis.

[20] Shibata N, Hayashi T, Fukumura R, Fujii J, Kudome-Takamatsu T, Nishimura O (2012). Comprehensive gene expression analyses in pluripotent stem cells of a planarian, *Dugesia japonica*. Int J Dev Biol.

[21] Rouhana L, Weiss JA, Forsthoefel DJ, Lee H, King RS, Inoue T (2013). RNA interference by feeding in vitro-synthesized double-stranded RNA to planarians: methodology and dynamics. Dev Dyn.

[22] Tazaki A, Gaudieri S, Ikeo K, Gojobori T, Watanabe K, Agata K (1999). Neural network in planarian revealed by an antibody against planarian synaptotagmin homologue. Biochem Biophys Res Commun.

[23] Sakai F, Agata K, Orii H, Watanabe K (2000). Organization and regeneration ability of spontaneous supernumerary eyes in planarians -eye regeneration field and pathway selection by optic nerves. Zool Sci.

[24] Nishimura K, Kitamura Y, Inoue T, Umesono Y, Yoshimoto K, Taniguchi T (2008). Characterization of tyramine beta-hydroxylase in planarian *Dugesia japonica*: cloning and expression. Neurochem Int.

[25] Weimer BR (1918). Thigmotactic Reactions of the Fresh Water Turbellarian, *Phagocata gracilis*, Leidy. Trans Am Microsc Soc.

[26] Miyamoto S, Shimozawa A (1985). Chemotaxis in the Freshwater Planarian, *Dugesia japonica japonica*. Zool Sci.

[27] Pigon A, Morita M, Best JB (1974). Cephalic mechanism for social control of fissioning in planarians. II. Localization and identification of the receptors by electron micrographic and ablation studies. J Neurobiol.

[28] Nishimura K, Kitamura Y, Umesono Y, Takeuchi K, Takata K, Taniguchi T (2008). Identification of glutamic acid decarboxylase gene and distribution of GABAergic nervous system in the planarian *Dugesia japonica*. Neuroscience.

[29] Thompson R, McConnell JV (1955). Classical conditioning in the planarian, *Dugesia dorotocephala*. J Comp Physiol Psychol.

[30] Hartry AL, Keith-Lee P, Morton WD (1964). Planaria: Memory Transfer through Cannibalism Reexamined. Science.

[31] Rilling M (1969). The mystery of the vanished citations: James McConnell's forgotten 1960s quest for planarian learning, a biochemical engram, and celebrity. American Psychologist.

[32] Shomrat T, Levin M (2013). An automated training paradigm reveals long-term memory in planarians and its persistence through head regeneration. J Exp Biol.

[33] Prados J, Alvarez B, Howarth J, Stewart K, Gibson CL, Hutchinson CV (2013). Cue competition effects in the planarian. Anim Cogn.

[34] Asano Y, Nakamura S, Ishida S, Azuma K, Shinozawa T (1998). Rhodopsin-like proteins in planarian eye and auricle: Detection and functional analysis. J Exp Biol.

[35] Zamanian M, Kimber MJ, McVeigh P, Carlson SA, Maule AG, Day TA (2011). The repertoire of G protein-coupled receptors in the human parasite *Schistosoma mansoni* and the model organism *Schmidtea mediterranea*. BMC Genomics.

[36] Nishimura K, Kitamura Y, Inoue T, Umesono Y, Sano S, Yoshimoto K (2007). Reconstruction of dopaminergic neural network and locomotion function in planarian regenerates. Dev Neurobiol.

[37] Nishimura K, Kitamura Y, Taniguchi T, Agata K (2010). Analysis of motor function modulated by cholinergic neurons in planarian *Dugesia japonica*. Neuroscience.

