# Supplementary figures and images for: Planarian shows decision-making behavior in response to multiple stimuli by integrative brain function
Source: Zoological Lett. 2015 Feb 1;1:7. doi: 10.1186/s40851-014-0010-z (PMC4657317; doi:10.1186/s40851-014-0010-z)

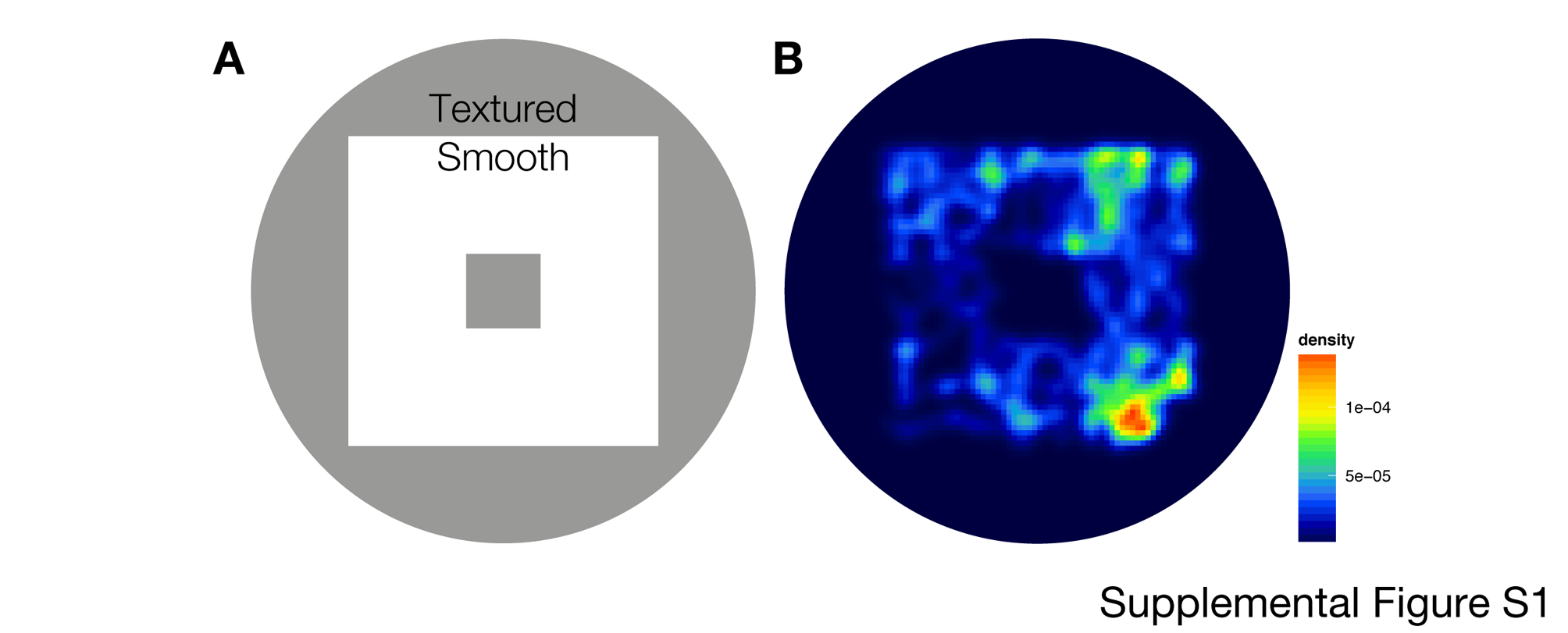

Supplement: Additional file 1: Figure S1. — Planarian behaviors in arbitrary smooth surface region. (A) Schematic drawing of textured dish for thigmotaxis/kinesis assay. Textured regions are indicated by gray color. The other region (colored white) indicates a smooth region. (B) Heat map view of planarians’ movement. Planarians only moved on the smooth surface region, although planarians usually tend to move near the edge of a dish as a default behavior. [file 40851_2014_10_MOESM1_ESM.tiff]
